# Development of a Label-Free Electrochemical Aptasensor for the Detection of Tau381 and its Preliminary Application in AD and Non-AD Patients’ Sera

**DOI:** 10.3390/bios9030084

**Published:** 2019-06-30

**Authors:** Dan Tao, Bingqing Shui, Yingying Gu, Jing Cheng, Weiying Zhang, Nicole Jaffrezic-Renault, Shizhen Song, Zhenzhong Guo

**Affiliations:** 1 Hubei Province Key Laboratory of Occupational Hazard Identification and Control, Medical College, Wuhan University of Science and Technology, Wuhan 430065, China; 2 Resources and Environmental Engineering College, Wuhan University of Science and Technology, Wuhan 430081, China; 3 Key Laboratory of Optoelectronic Chemical Materials and Devices of Ministry of Education, Institute for Interdisciplinary Research, Jianghan University, Wuhan 430056, China; 4 University of Lyon, Institute of Analytical Sciences, UMR-CNRS 5280, 5, La Doua Street, 69100 Villeurbanne, France

**Keywords:** aptasensor, tau381, carboxyl graphene/thionin/gold nanoparticles, human serum, Alzheimer′s disease

## Abstract

The electrochemical aptamer sensor has been designed for detecting tau381, a critical biomarker of Alzheimer′s disease in human serum. The aptasensor is obtained by immobilizing the aptamer on a carboxyl graphene/thionin/gold nanoparticle modified glassy-carbon electrode. As a probe and bridge molecule, thionin connected carboxyl graphene and gold nanoparticles, and gave the electrical signal. Under optimal conditions, the increment of differential pulse voltammetry signal increased linearly with the logarithm of tau381 concentration in the range from 1.0 pM to 100 pM, and limit of detection was 0.70 pM. The aptasensor reliability was evaluated by determining its selectivity, reproducibility, stability, detection limit, and recovery. Performance analysis of the tau381 aptasensor in 10 patients’ serum samples showed that the aptasensor could screen patients with and without Alzheimer′s disease. The proposed aptasensor has potential for use in clinically diagnosing Alzheimer′s disease in the early stage.

## 1. Introduction

Patients of Alzheimer’s disease (AD) in the world were estimated to be approximately fifty million in 2018, a number that is similar to the population of Kenya, Colombia, or Korea. It will increase by threefold in 2050. At present, the annual cost of the disease is about $1 trillion, and it is estimated that the cost will be doubled by 2030 [1,2,3,4]. The outcome of AD is irreversible damage to brain cells, and AD has become a serious medical problem globally, as early screening is the only way to reduce trouble when there are no drugs available. β-amyloid and tau protein are considered to both be major proteins that form the cause of AD. Tau381 (1N3R), one of tau protein’s six subtypes, has a crucial role as a critical biomarker in the early diagnosis of AD. Using the tau-PET tracer [^18^F] THK5317, [(S)-[^18^F] THK5117] and [^18^F] FDG in a human cerebrospinal fluid (CSF) sample of AD patients was investigated by Leuzy et al. [5] and their study found that a subtype of tau may better capture tau pathology and synaptic impairment. Mielkea et al. reported the tau protein result of blood was in accordance with CSF and illustrated blood tau protein as a clinically useful biomarker [6].

Aptamers are short ssDNA or ssRNA nucleic acid molecules synthesized in vitro that specifically pair with proteins, nucleic acids, viruses, bacteria, parasites, and other targets [7,8]. They can fold into proper three-dimensional structures via specific matching between complimentary strands, electrostatic interactions, and hydrogen bonds, etc. Aptamers can be invoked as recognition elements superior to antibodies because of their chemical stability, ease to modify, high affinity, specificity, and fixation density on the electrodes. Krylova et al. [9] used affinity-mediated non-equilibrium capillary electrophoresis of equilibrium mixtures to study the interaction of tau381 and aptamers, and the dissociation rate constant K*d* was 0.19 ± 0.01 μM; moreover, the K*d* value found by Lisi et al. [10] was 116 ± 6 nM. Thus, aptamers are particularly attractive in the field of clinical testing and electrochemical sensing [11,12]. Various biosensor transductions are displayed in Table 1, fluorescence [10], electrochemistry [13,14,15,16], and surface plasmon resonance (SPR) [17,18,19], have been developed for tau biosensors. Until now, the electrochemical transduction was associated with anti-tau antibody as a recognition element.

So far there is a wide utilization of graphene and graphene-related nanomaterials in sensors and medical science [21,22,23,24] due to their small size, excellent biocompatibility, excellent conductivity, ease to be functionalized, high specific surface area, and mechanical strength [25,26,27]. Carboxyl graphene (CG) as a low-dimensional carrier for the growth of gold nanoparticles (Au NPs) enhances the electronic properties of graphene because of their space constraints and synergistic interactions. The sensitivity can be greatly improved when they are combined with electrochemical detection [28].

**Figure 1:**
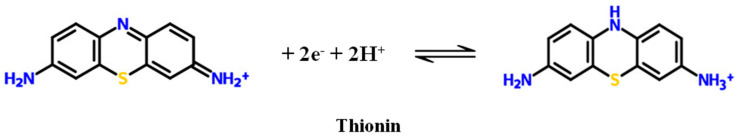
The structure and reversible reaction of thionin.

Thionin (TH) is a phenothiazine cationic dye with conjugate ring plane structure, which is composed of two benzene rings, two amino groups, and one heterocyclic ring. TH has the properties of electrochemical reversibility, stability, and fast electron transfer as a redox probe and bridging molecule (Figure 1).

**Scheme 1:**
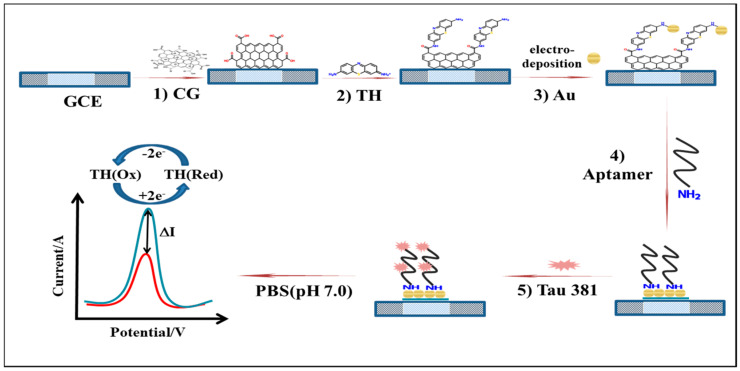
Preparation of glassy-carbon electrode/carboxyl graphene/thionin/gold nanoparticles/aptamer/tau381 aptasensor. 1) doped carboxyl graphene on a glassy-carbon electrode surface, 2) followed by incubation with thionin, 3) electro-deposition and reduction of HAuCl_4_·3H_2_O to gold nanoparticles, then incubation with aptamer 4) and tau381 5); finally, differential pulse voltammetry evaluation of tau381 levels in human serum.

We propose to include this redox probe directly in the bionanocomposite that applied to the glassy-carbon electrode (GCE) surface. As illustrated in Scheme 1, we fabricated Au-TH-CG-aptamer as the bio-recognition element for capturing the tau381. CG was electrostatically adsorbed upon the surface of the clean GCE; then TH was conjugated to CG through π-stacking and condensation interactions [29,30]. The Au NPs were formed by electrodeposition. The amine modified tau381-aptamer interacts with Au NPs through Au-N and hydrogen bonds. The Au-TH-CG nanocomposite was prepared more quickly (less than 1 hour), without reflux, centrifugation, etc. Finally, the tau381-aptamer quickly recognized tau381 through its specific affinity.

The electrochemical signal of thionin is recorded by differential pulse voltammetry (DPV) and represents the response of this label-free aptasensor. The new design scheme was further tested for the actual clinical detection of tau381.

## 2. Experimental

### 2.1. Reagent and Chemicals

The 5 mg/ml CG solution (Nanjing XFNANO Materials Tech Co., Ltd., Nanjing, China) was diluted to 80 μg/ml and then stored at 4 °C. TH and HAuCl_4_·3H_2_O were obtained by Sigma-Aldrich. Recombinant human tau381 protein (Abcam, Cambridgeshire, UK) was prepared into each concentrations of solution to be measured. The ssDNA-aptamer sequence [9] was 5′-GCGGAGCGTGG CAGG-3′, and the 5′-H_2_N- modified aptamer was synthesized by Sangon Biotech Co., Ltd. and purified by high performance liquid chromatography. 5 μM aptamer stock solution was prepared in 0.1M phosphate buffer saline (PBS, pH 7.4) and stored at −20 °C.

### 2.2. Apparatus

The prepared electrodes were characterized by field emission scanning electron microscopy (Zeiss SIGMA, Cambridge, UK). Cyclic voltammetry (CV), electrochemical impedance spectroscopy (EIS), and differential pulse voltammetry (DPV) were performed on a CHI 660E electrochemical Analyzer (CH Instruments, Shanghai, China). 3 mm diameter GCE or Au-TH-CG/GCE working electrode was combined with Pt-wire auxiliary electrode and saturated Ag/AgCl reference electrode into a three-electrode system.

### 2.3. Fabrication of Carboxyl Graphene/Thionin/Gold Nanoparticles Nanocomplex

Before modifying, the GCE was ultrasonic wave-cleaned in ethanol and double distilled water for a few mins, after being polished with alpha-alumina powder of 0.3 and 0.05 µm, then dried under nitrogen (N_2_) flow. Then 7.5 μL of CG dispersion that was previously ultrasonicated for half an hour was added to the electrode surface, the removal of excess moisture took place under an infrared light and was washed with deionized water. After nitrogen blowing, the electrode was incubated with 12.5 μL aqueous solution of TH (0.5 mM) and naturally dried [31]. Next, to form the AuNPs to the electrode, TH–CG/GCE was immersed in 0.2 mg/mL HAuCl_4_ for electrodeposition with cyclic sweeping [32]. The obtained Au-TH–CG/GCE was rinsed with distilled water and removed water and oxygen under N_2_ conditions. After that, 20 μL of 5 μM aptamer solution was dropped on the surface of the Au-TH–CG/GCE, then remained at 4 °C overnight. Finally, the excess of bio- recognition element was washed out carefully with 0.1 M PBS (pH 7.4), dried under N_2_ atmosphere, and the aptasensor was stored into the refrigerator for backup.

### 2.4. Electrochemical Detection of Tau381

The required concentration of tau381 (20 μL) was captured on the aptasensor and kept for 30 min at room temperature. Then, the electrode was washed with 0.1 M PBS (pH 7.4) to remove the non-specific binded tau381. DPV was recorded in PBS (pH 7.0) at a scanning range from −0.6 V to 0.2 V to obtain an electrochemical response. The electrode surface was gently rinsed with 0.1 M PBS (pH 7.4) and dried under N_2_ gas for measuring the next concentration.

### 2.5. Human Serum and Ethics

A total of 10 serum samples were collected by the community service center of Dongbao District in Jingmen. All subjects were over 65 years old (10 subjects divided into non-AD patient and AD patient groups). According to NINCDS-ADRDA Alzheimer’s Criteria, patients have undergone multiple neurological examinations in the CHSC and have been clinically diagnosed with AD.

This research was approved by the ethics committees of the community service center of Dongbao District and the Wuhan University of Science and Technology, which are consistent with the Helsinki Declaration, and all subjects were informed and gave agreement to participating in this investigation in advance.

## 3. Results and Discussion

### 3.1. Morphological Characterization of the Carboxyl Graphene/Thionin/Gold Nanoparticles Electrode Surface

**Figure 2:**
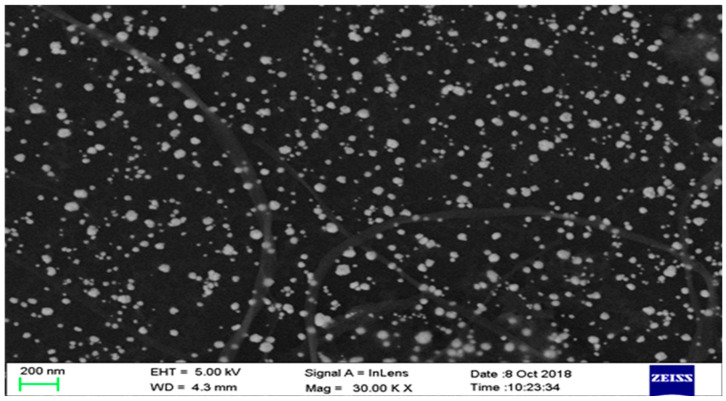
Field emission scanning electron microscopy image of Au NPs-TH-CG on GCE.

The morphologies of the prepared CG-TH-Au NPs composite on the GCE were characterized by field emission scanning electron microscope. It is easy to see that in Figure 2, the dark areas of the image are a layer of CG folds, and homogeneously distributed spherical particles of AuNPs around 50 nm in diameter. The AuNPs, on the one hand, increase the electrochemical activity; on the other hand, they provide a specific binding surface for aptamers [33,34].

### 3.2. Electrochemical Characterization of the Aptasensor

**Figure 3:**
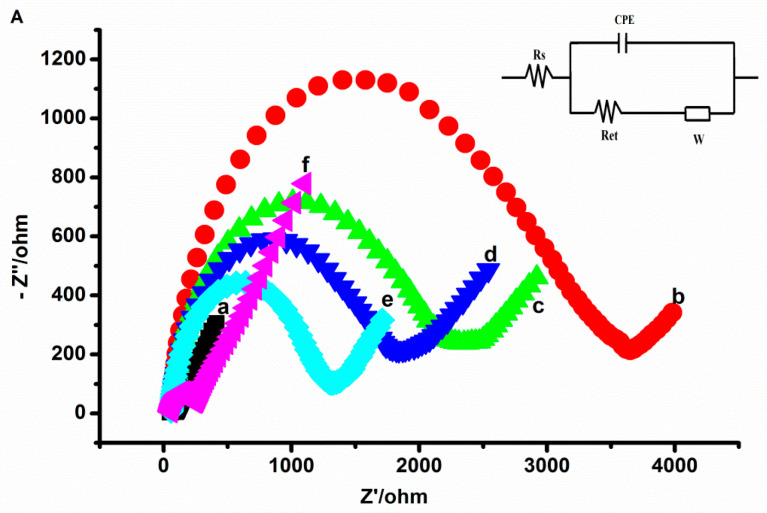

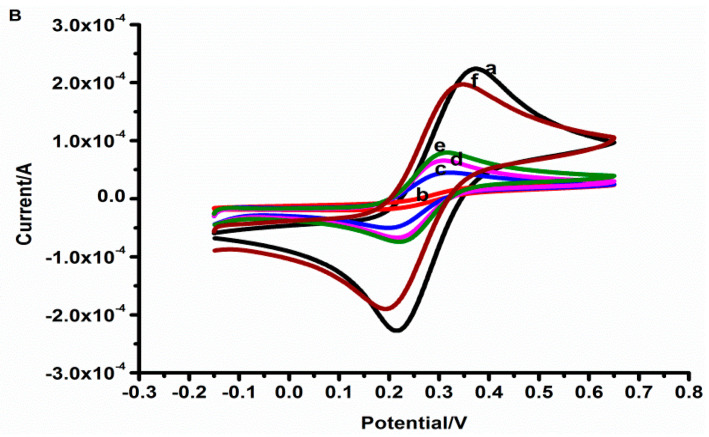
(**A**) Impedance plots of: bare electrode (a), CG/GCE (b), TH-CG/GCE (c), Au-TH-CG/GCE (d), Apt-Au-TH-CG/GCE (e), tau381-Apt-Au-TH-CG/GCE (f) in 10 mM [Fe(CN)6]^3−/4–^ (1:1 ratio). In the inset, Rs, W, Ret, and CPE stand for the solution resistance, the Warburg diffusion resistance, the electron-transfer resistance, and the double layer capacitance, respectively. (**B**) Cyclic voltammetry of Fe(CN)6]^3−/4−^ for: bare electrode (a), CG/GCE (b), TH-CG/GCE (c), Au-TH-CG/GCE (d), Apt-Au-TH-CG/GCE (e), tau381-Apt-Au-TH-CG/GCE (f) in 0.1M PBS (pH 7.0) containing 10 mM [Fe(CN)6] ^3−/4–^ (1:1 ratio).

For electrochemical surface features of the fabrication steps of the aptasensor, EIS and CV were performed in 10 mM [Fe (CN)_6_]^3−/4−^. The Nyquist plots of the measured EIS data are shown in Figure 3A. The smallest semi-circle of bare GCE (curve a) can be covered, but the straight line was seen very clearly, which indicated that bare GCE presents relatively low impedance and good conductivity. Compared to bare GCE, the electron transfer resistance (Ret) increased greatly when CG was adsorbed on the GCE surface (curve b), owing to the GCE electron transfer hindrance by a dense CG film. Subsequently, the CG/GCE surface was modified with TH, the semi-circle was smaller than curve b (curve c) on account of the electronic properties of TH. After AuNPs were combined to the TH-CG/GCE, the Ret decreased again (curve d), showing the AuNPs could improve the current transmission rate. The Ret significantly decreased when the tau381-aptamer was adsorbed (curve e), the amino-modified aptamers were bound to the surface of the AuNPs. The specific recognition between aptamer and tau381 (curve f) increases the electron transfer rate.

In Figure 3B, the peak currents of [Fe(CN)_6_]^3−/4–^ at differently treated electrodes followed the increased trend of TH-CG/GCE(c) <Au-TH-CG/GCE (d) <Apt-Au-TH-CG/GCE (e) <tau381-Apt-Au-TH-CG/GCE (f). The CV results verified that in the presence of TH, the charge transfer rate is highly improved, which is in agreement with the EIS results.

### 3.3. Optimization of Effective Parameters for Aptasensor Response

To acquire the ideal results for the aptasensor, the influence factors (e.g., pH value, aptamer concentration, incubating time, and serum dilution ratio) were optimized.

**Figure 4:**
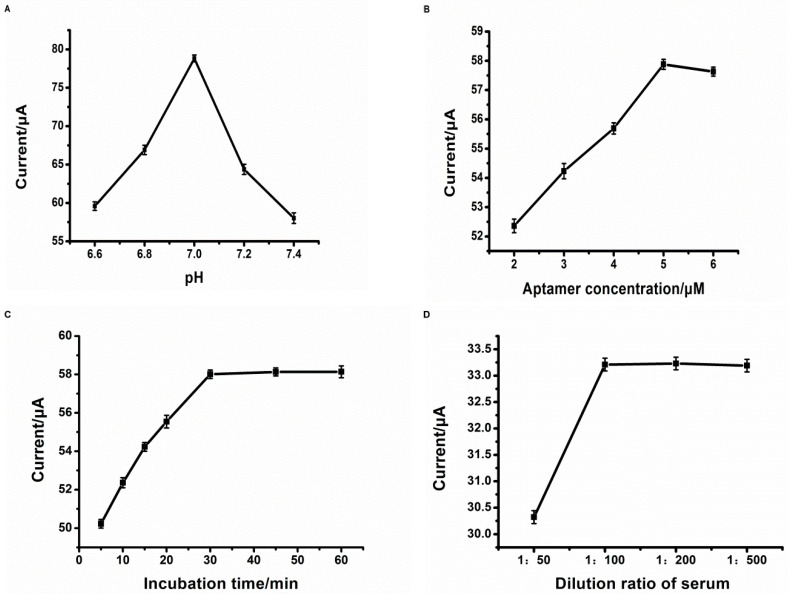
Optimization of experimental conditions for detection of tau381. pH value (**A**): 6.6, 6.8, 7.0, 7.2, and 7.4; aptamer concentration; (**B**): 2, 3, 4, 5, and 6 μM; aptamer-tau381 incubating time; (**C**): 5, 10, 15, 20, 30, 45, and 60 min; dilution ratio of serum; (**D**): 1:50, 1:100, 1:200, and 1:500, respectively. The concentration of tau381 was 20 pM and the current signal was obtained by the DPV which was carried out in 0.1 M PBS (pH 7.0) from −0.6 to 0.2 V.

Current responses increased rapidly with the increasing pH value (Figure 4A) from 6.6 to 7.0 and then decreased steeply from 7.0 to 7.4; hence, pH 7.0 was selected as the optimal pH value. At the optimized pH value, the amperometric responses increased with aptamer concentration (Figure 4B) and then tended to constant values after 5 μM, suggesting that saturation of the tau381-aptamer occurs. As the excess tau381-aptamer could block electron transport, a higher aptamer concentration decreased the response. Therefore, subsequent experiments employed 5 μM as the best concentration for all the incubation steps of the assay.

Figure 4C showed the effect of the time of conjunct aptamer and tau381 on the current response of aptasensor. The current response value increased as the incubation time increased from 5 min to 30 min, and the current signal value no longer changes after 30 minutes, which showed a saturated conjugate between tau381 and aptamer. As the incubation time was over 30 min, the current signal approached a constant value. Consequently, the incubating time was chosen to be 30 min for all experiments.

The dilution ratio was also optimized on the basis of preliminary work of our research group [20]. In Figure 4D, the current responses increased with the increasing dilution ratio of serum from 1:50 to 1:100, the amperometric responses that remained changed a few over 1:100. Thus, the dilution ratio was chosen to be 1:100. When the serum was not diluted enough, fouling of the electrode led to a decrease of the electrochemical signal.

### 3.4. Determination of Tau381

**Figure 5:**
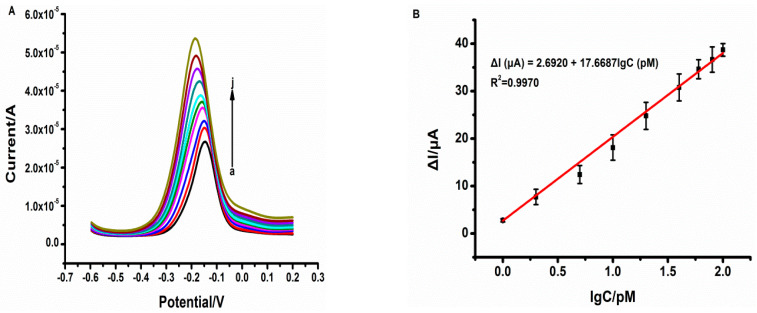
(**A**) Differential pulse voltammetry curves of tau381 concentrations including 0, 1, 2, 5, 10, 20, 40, 60, 80, and 100 pM in 0.1 M PBS (pH 7.0); (**B**) Plot of ΔI vs. Log of tau381 concentration (n > 3).

TH can act as an electrochemical redox probe because of its electrochemical reversibility properties, i.e., (TH (Ox) + 2e^−^ ⇌ TH (Rex) was shown in Figure 1). For increasing concentration of tau381, the reduction peak of TH was observed to increase, which allows verifying of the specific binding ability of tau381 with tau381-aptamer (Figure 5A). The variation of DPV current intensity (ΔI) of aptamer-Au-TH–CG/GCE was used for quantitative detection of tau381.

Under the optimized parameters, the measurement signal (ΔI) increased linearly with the logarithm of concentration of tau381 from 1 to 100 pM, and the linear curve fits a regression equation of ΔI (μA) = 2.6920 + 17.6687lgC (pM) with a correlation coefficient of 0.9985, as seen in Figure 5B. The limit of detection (LOD), calculated at a signal/noise ratio of 3, was found to be 0.70 pM. The proposed label-free aptasensor showed a slightly higher detection limit compared to that of sandwich-based tau381 immunosensors [14,20].

### 3.5. Selectivity, Reproducibility, and Stability

**Figure 6:**
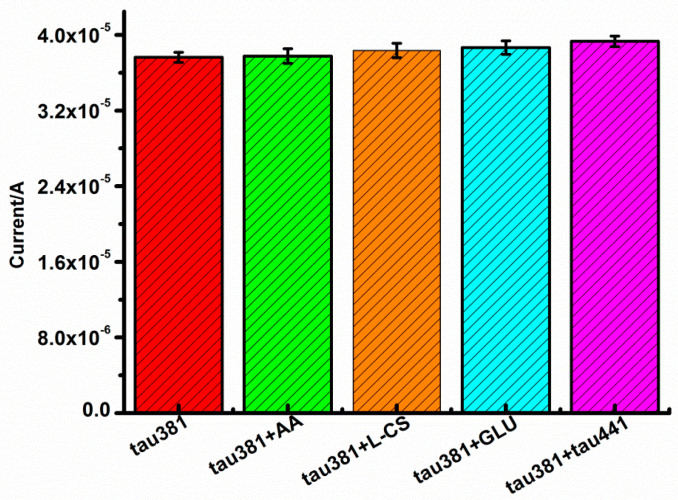
Current response of the aptasensor to 1 pM tau381, 1 pM tau381 + 100 pM AA, 1 pM tau381 + 100 pM L-CS, 1 pM tau381 + 100 pM GLU, 1 pM tau381 + 100 pM tau441 (n > 3).

To test the selectivity of the aptasensor, ascorbic acid, L-cysteine, glucose, and tau441 were chosen as the potentially interfering substances, the 1 pM tau381 solution containing 100 pM interfering substances. The responses of interfering substances, shown in Figure 6, are 2.1%, 2.0%, 1.8%, and 1.4%, respectively (less than 5%), lower than that without interfering substance. Results of the interference test showed that the selectivity of this aptasensor was acceptable.

Furthermore, the reproducibility of this aptasensor was tested. Five fresh electrodes prepared independently were employed for the detection of tau381 with the same concentration (10 pM). The relative standard deviation (RSD) of the measurements was 4.9%, indicating that the aptasensor presents excellent inter-sensor repeatability for tau381 detection.

**Figure 7:**
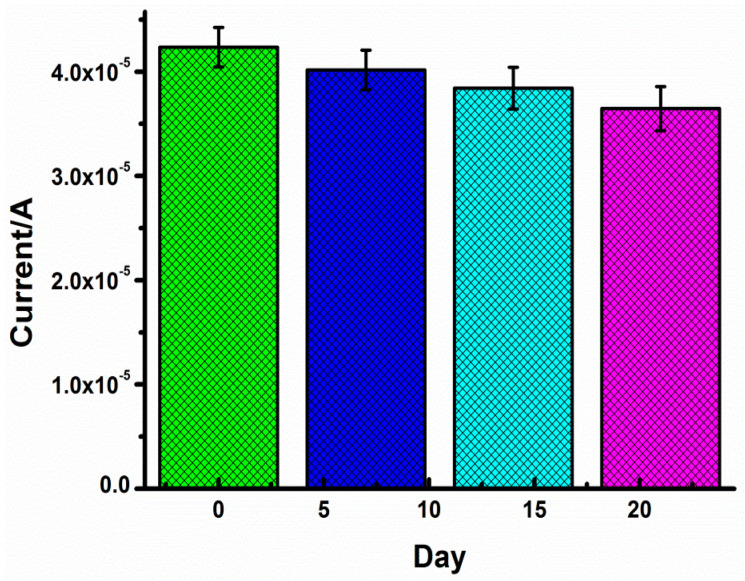
Aptamer-Au-TH–CG/GCE storage at 4 °C for 0, 7, 14, and 21 days with 10 pM tau381 (n > 3).

In addition, the stability was also checked in the 7th, 14th, and 21st days of storage at 4 °C, separately (Figure 7). The activity of the aptasensor can be maintained respectively at 94.88%, 90.76%, and 86.12%, showing high stability of the aptasensor. In other words, we can think the quality guarantee period of the aptamer-sensor is 21 days.

### 3.6. Application in Analysis of Human Serum Samples

To demonstrate the reliability of the proposed aptasensor for real sample analysis, three human serum samples were diluted 100 times for detection. The recovery was used for determination by standard addition methods. The results from Table 2: recovery ranged from 99.1% to 101.0% and the RSDs ranged from 1.08% to 4.07%, showing that the proposing method is acceptable.

### 3.7. Analysis Performance of Tau381 Aptasensor in Non- and Alzheimer′s Disease Patient Samples

**Figure 8:**
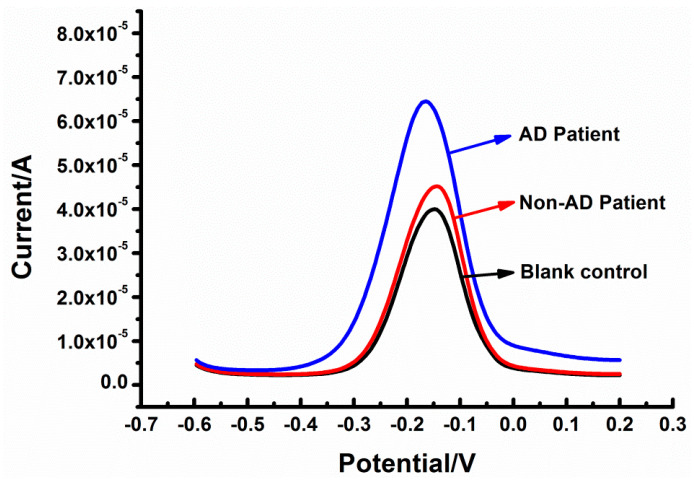
The dpvs of tau381 in human serum samples from blank control, non- and Alzheimer′s disease patients.

Figure 8 shows the current response of a blank control (a young man’s serum), a Non-AD patient, and an AD patient (*I*_Blank control_ < *I*_Non-AD patient_ < *I*_AD patient_). In Table 3, we chose five Non-AD patients and AD patients. Their tau381 levels are represented by the mean value ± SD. The detection contents of Non-AD patients ranged from 100 ± 6 pM to 520 ± 74 pM, and AD patients ranged from 1778 ± 86 pM to 4612 ± 62 pM, suggesting that the aptasensor could screen patients with AD and Non-AD, in excellent agreement with the results from an ELISA kit.

## 4. Conclusions

In summary, we developed a label-free electrochemical aptasensor of CG/TH/AuNPs for detecting tau381 protein successfully. The electrochemical response is based on the TH-based DPV response increment before and after the incubation of tau381. Combining CG, TH, AuNPs, and tau381 aptamer, the proposed aptasensor displayed excellent performance with good selectivity, stability, and reproducibility. In addition, a readily achievable detection limit of 0.70 pM with a linear range from 1 pM to 100 pM was obtained. The aptasensor could screen the AD patients, therefore allowing early diagnosis.

## Figures and Tables

**Table 1:** A comparison of different biosensor techniques for tau detection.

Techniques	Modified	Linear Rang	LOD	Target	Ref.
Fluorescence	FAM/ssDNA	-	28 nM	Tau441	[10]
SPR	aptamer/tau/anti-tau/ MUA	10–100 fM	10 fM	Tau381	[17]
	tau/mAb/protein G	0.01–100 ng/mL	10 pg/mL	Tau441	[18]
	tau/anti-tau/11-MUA	10–2000 pg/mL	2.4 pg/mL	T-tau	[19]
Electrochemistry	tau/anti-tau/pPG/GO	0.5–15.1 nM	0.15 nM	T-tau	[13]
	tau/anti-tau/protein G/ DTSSP	10^−14^–10^−7^ M	0.03 pM	Tau441	[14]
	CS-Au-Aptamer/tau/anti-tau/MPA	0.5–100.0 pM	0.42 pM	Tau381	[20]
	tau/tau	0.2–1.0 μM	-	Tau441	[15]
	tau/anti-tau/MPA- SAM	10^3^–10^5^ pg/mL	-	T-tau	[16]

FAM: 5-Carboxyfluorescein; MUA: 11-mercaptoundecanoic acid; pPG: poly (propyleneglycol); GO: graphene oxide; DTSSP: 3, 3’-dithiobis (sulfosuccinimidyl propionate); CS: cysteamine; MPA-SAM: Self-assembled monolayers of 3-mercaptopropionic acid.

**Table 2:** The detection of tau-381 in human serum samples.

Sample No.	Initial Concentration (pM)	Add (pM)	Detection (pM)	Recovery (%)	RSDs (%)
1	0.75	2.00	2.80, 2.77, 2.78, 2.72, 2.78	101.0	1.08
2	0.75	20.00	19.68, 22.03, 20.56, 20.89, 21.01	100.4	4.07
3	0.75	60.00	58.36, 59.36, 59.23, 60.66, 62.12	98.6	2.45

**Table 3:** Assay results of clinical serum samples using aptasensor and ELISA Kit.

	Sample No.	Content Detected with the Aptasensor (Mean ± SD, pM)	Content Detected with ELISA Kit (Mean ± SD, pM)
Non-AD Patient	1	100 ± 6	115 ± 41
	2	163 ± 58	128 ± 26
	3	520 ± 74	553 ± 31
	4	248 ± 62	274 ± 26
	5	269 ± 67	295 ± 38
AD Patient	1	1778 ± 86	1833 ± 58
	2	4022 ± 59	4057 ± 54
	3	3883 ± 61	3923 ± 42
	4	4612 ± 62	4730 ± 51
	5	3558 ± 55	3666 ± 45
