# Correction: Tao et al. Development of a Label-Free Electrochemical Aptasensor for the Detection of Tau381 and its Preliminary Application in AD and Non-AD Patients’ Sera. *Biosensors* 2019, *9*, 84

**DOI:** 10.3390/bios15040234

**Published:** 2025-04-07

**Authors:** Dan Tao, Bingqing Shui, Yingying Gu, Jing Cheng, Weiying Zhang, Nicole Jaffrezic-Renault, Shizhen Song, Zhenzhong Guo

**Affiliations:** 1 Hubei Province Key Laboratory of Occupational Hazard Identification and Control, Medical College, Wuhan University of Science and Technology, Wuhan 430065, China; tstower@hotmail.com (D.T.);; 2 Resources and Environmental Engineering College, Wuhan University of Science and Technology, Wuhan 430081, China; 3 Key Laboratory of Optoelectronic Chemical Materials and Devices of Ministry of Education, Institute for Interdisciplinary Research, Jianghan University, Wuhan 430056, China; 4 University of Lyon, Institute of Analytical Sciences, UMR-CNRS 5280, 5, La Doua Street, 69100 Villeurbanne, France

## Text Correction

In the original publication [1], a correction has been made to Section 3.7, Paragraph 1. The revised version is as follows: 

Figure 8 shows the current response of a blank control (a young man’s serum), a Non-AD patient, and an AD patient (*I*_Blank control_ < *I*_Non-AD patient_ < *I*_AD patient_). In Table 3, we chose five Non-AD patients and AD patients. Their tau381 levels are represented by the mean value ± SD. The detection contents of Non-AD patients ranged from 100 ± 6 pM to 520 ± 74 pM, and AD patients ranged from 1778 ± 86 pM to 4612 ± 62 pM, suggesting that the aptasensor could screen patients with AD and Non-AD, in excellent agreement with the results from an ELISA kit.

## Error in Table

In the original publication [1], there was a mistake in Table 3 as published. When two different methods were used to test the same batch of samples, the data obtained were identical. Subsequently, we carefully verified the data and corrected Table 3 based on the feedback from the Editorial Office. The corrected Table 3 appears below. 

The authors state that the scientific conclusions are unaffected. This correction was approved by the Academic Editor. The original publication has also been updated.

## Figures and Tables

**Table 3:** Assay results of clinical serum samples using aptasensor and ELISA Kit.

	Sample No.	Content Detected with the Aptasensor (Mean ± SD, pM)	Content Detected with ELISA Kit (Mean ± SD, pM)
Non-AD Patient	1	100 ± 6	115 ± 41
	2	163 ± 58	128 ± 26
	3	520 ± 74	553 ± 31
	4	248 ± 62	274 ± 26
	5	269 ± 67	295 ± 38
AD Patient	1	1778 ± 86	1833 ± 58
	2	4022 ± 59	4057 ± 54
	3	3883 ± 61	3923 ± 42
	4	4612 ± 62	4730 ± 51
	5	3558 ± 55	3666 ± 45
